# Detection and treatment of transplant renal artery stenosis

**DOI:** 10.4103/0970-1591.45538

**Published:** 2009

**Authors:** Sriram Krishnamoorthy, Ganesan Gopalakrishnan, Nitin Sudhakar Kekre, Ninan Chacko, Shyam Keshava, George John

**Affiliations:** Department of Urology, Christian Medical College, Vellore, India; 1 Department of Radiology, Christian Medical College, Vellore, India; 2 Department of Nephrology, Christian Medical College, Vellore, India

**Keywords:** Angioplasty, peak systolic velocity, transplant renal artery stenosis

## Abstract

**Purpose:**

To assess the effects of transplant renal artery stenosis (TRAS) on blood pressure, renal function, and graft survival. To assess the usefulness of Doppler in predicting the clinical significance of TRAS and also to identify the predictive factors in Doppler that correlated with clinical features of TRAS.

**Materials and Methods:**

A prospective study was done on consecutive renal allograft recipients at Christian Medical College, over a period of 66 months from January 2002. All recipients underwent Doppler ultrasound (DUS) evaluation on the fifth post-operative day. Subsequent evaluation was done if the patients had any clinical or biochemical suspicion of TRAS. Angiogram was done in case of a high index of suspicion of significant stenosis or before angioplasty and stenting. The clinical and radiological outcomes of the patients with symptomatic or asymptomatic TRAS were analyzed.

**Results:**

Five hundred and forty three consecutive renal allograft recipients were analyzed, of whom, 43 were found to have TRAS. Nine recipients (21%) were detected to have TRAS on first evaluation. All had a high peak systolic velocities (PSV) recorded while 25 of them had other associated features. Patients with only high PSV required no further intervention and were followed up. They had a pretransplant mean arterial pressure (MAP) of 107.83 mmHg (SD = 13.32), ranging from 90 to 133 mm Hg and a posttransplant MAP of 106.56 mmHg (SD =16.51), ranging from 83 to 150 mm Hg. Their mean nadir serum creatinine was 1.16 mg% (SD = 0.24), at detection was 1.6 mg% (SD = 1.84) and at 6 months follow-up was 1.26 mg% (SD=0.52). Of the remaining 25 patients with other associated Doppler abnormalities, 11 required further intervention in the form of re-exploration in 2, angioplasty in 3 and stenting in 6 patients. One patient in the group of patients intervened, expired in the immediate post-operative period due to overwhelming urosepsis and consumption coagulopathy. The mean creatinine clearance (Cockroft-Gault method) in this group of remaining 10 patients, before and after intervention was 44.75 ml/min (SD=17.85) and 68.96 ml/min (SD = 10.56), respectively, with a mean increase by 24.21 ml/min (*P*=0.000). The mean arterial pressure before and after intervention in this group were 132.80 mm Hg (SD = 13.22) and 102 mm Hg (SD = 10.55), with a decline in the MAP by 30.80 mmHg (*P*=0.017). The haemoglobin levels also increased from 11.72 (SD=2.13) to 12.48 gm% (SD = 1.75), with a mean increase by 0.76 gm% (*P*=0.05).

**Conclusions:**

Patients with isolated high PSV do not have a significant alteration of blood pressure or allograft function and required no intervention. Although high PSV with associated Doppler anomalies are more suggestive of significant TRAS, the decision regarding surgical intervention is largely based on clinical assessment.

## INTRODUCTION

Hypertension is a common problem after renal transplantation. About 50-60% of recipients with long-term functioning grafts develop hypertension.[1] The aetiology could be multifactorial, including acute and chronic rejection, effects of drugs (corticosteroids and cyclosporine), native kidney disease and transplant renal artery stenosis (TRAS). TRAS is one of the potentially reversible causes of hypertension.

The overall reported incidence in literature varies from 1.5 to 23%.[2] This wide variation is mainly due to various factors.

First, the definition of haemodynamically significant TRAS has not been standardized. Various investigators have used a wide range of narrowing of the arterial lumen from 50% to greater than 80% in defining significant stenosis.[3] Second, the increasing use of Doppler ultrasound (DUS) and magnetic resonance angiography has led to an increased rate of detection of asymptomatic TRAS.[4]

TRAS is now recognized as a major cause of both graft dysfunction and loss. Early detection, appropriate treatment, and prevention of TRAS contribute significantly to patient and allograft survival.

Doppler evaluation has been used worldwide as a screening tool for the diagnosis of TRAS. This article mainly focuses on the clinical utility of DUS in the diagnosis of TRAS and also to assess the effects of TRAS on the allograft function.

## MATERIALS AND METHODS

We performed a prospective analysis of all consecutive renal allograft recipients between Jan 2002 and June 2007. Every patient who had symptomatic or radiologically detected findings suggestive of asymptomatic TRAS was included. Various factors that were associated with early detection of TRAS and the long-term effects of TRAS on blood pressures, renal function, and the ultimate allograft survival were analyzed.

The following data were recorded: native kidney disease, symptomatology at detection, time of detection, site of stenosis, presence or absence of rejection, immunosuppression used, blood pressure, haemoglobin levels and serum creatinine before and after transplantation, indications for and findings at Doppler ultrasonography and arteriography were analyzed.

The serum creatinine, haemoglobin levels and blood pressure before and after treatment of TRAS were compared using a paired two-tailed standard “t” test. The mean arterial pressure before and after transplant, number of anti hypertensive drugs required to control hypertension, serum creatinine at the time of detection and at 6 months follow up in the group of 18 patients with high peak systolic velocities (PSV) only were also compared using student “t” test. A P value of < 0.05 was considered to indicate a statistically significant difference.

As per our protocol, patients underwent a routine DUS evaluation on the fifth post-operative day by a senior radiologist with more than five years experience. Subsequent evaluation was done for those who developed uncontrolled hypertension requiring two or more drugs, delay in reaching nadir creatinine levels, oliguria or rising creatinine, proteinuria or history of pulmonary oedema.

TRAS was suspected when two or more criteria were present including (i) denovo or refractory hypertension, defined as an arterial pressure greater than 140/90 mmHg requiring two or more drugs; (ii) deterioration of renal function defined as greater than 20% increase in serum creatinine compared to the baseline (iii) sonographic abnormalities defined as PSV of more than 200 cm/s and/or a greater than 50% increase in PSV in any segment of the renal artery. PSV, location of TRAS, resistive index, and intra-segmental flow pattern were looked for in DUS. Angiogram was done in those with a strong clinical suspicion and/or a radiological suspicion of significant stenosis or before any form of intervention. Based on these criteria, 43 out of 543 renal allograft recipients were suspected to have TRAS and were included in our study. Symptomatic patients who had a significant stenosis or not responding to conservative treatment were subjected to reexploration, angioplasty and/or stenting.

## RESULTS

Of 543 consecutive renal allograft recipients analyzed over a period of five and a half years, 43 were diagnosed to have either clinical or radiological features of TRAS. The native kidney diseases in these patients were diabetic nephropathy in 8, adult polycystic kidney disease in 2, chronic glomerulonephritis in 2, one each of focal segmental glomerulosclerosis, IgA nephropathy, reflux nephropathy and neuro-vesical dysfunction. In 27 patients, the native kidney disease was unknown. Of these 43, two were from deceased donors.

**Figure 1:**
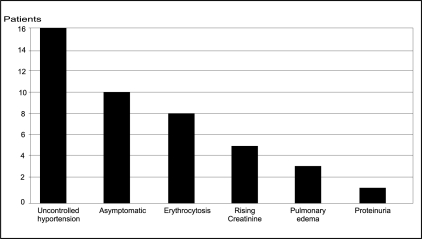
Mode of presentation

Figure 1 shows the various modes of presentation. More than three-fourths (76%) had either a clinical or biochemical abnormality suggestive of TRAS, while in 10 patients (24%), it was diagnosed during follow-up. These 10 asymptomatic patients had only a high PSV documented in DUS and were clinically and haemodynamically normal and did not require any further evaluation.

**Figure 2:**
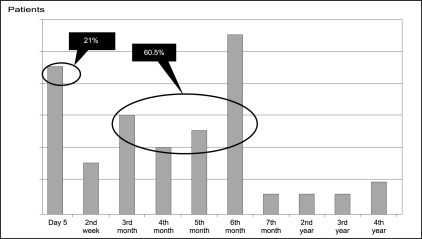
Time of detection of TRAS (n=43)

While 9 had a diagnosis made during routine day 5 Doppler evaluation, in 26 recipients, it was detected between 3 and 6 months postoperatively [Figure 2]. DUS evaluation alone was used in 26 (60%) of the patients to make a diagnosis of TRAS, while 17 required further imaging with angiogram either to confirm the severity of stenosis or prior to intervention.

Ten patients had double arteries. All 10 had the main artery anastomosed end to end to internal iliac artery. Of the accessory arteries, 5 were anastomosed end to side to external iliac artery, 2 were trousered and one was anastomosed to inferior epigastric artery. In two patients, the accessory arteries were very small and hence were ligated.

**Figure 3:**
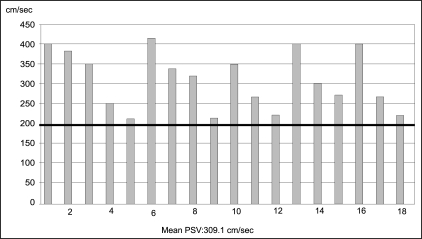
PSV of patients with an otherwise normal Doppler (n=18)

**Figure 4:**
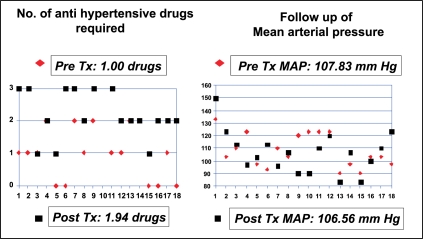
Follow up of patients with high PSV only (n=18)

**Figure 5:**
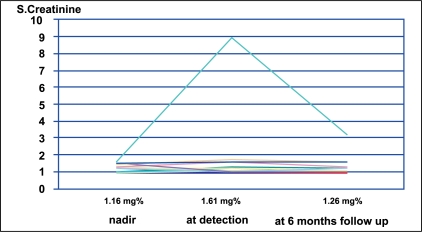
Follow up of serum creatinine in patients with high PSV (n=18)

Of the 43 patients, 18 had a detection of high PSV of more than 200 cm/s, with no evidence of stenosis or dampening of flow [Figure 3]. These patients were clinically stable with stable serum creatinine and blood pressures. In this group of 18 patients, even though the number of anti hypertensive drugs required after transplant was nearly double that was required pre-operatively, their average pre-transplant mean arterial pressure (MAP) was 107.83 mmHg (SD=13.32) and a post-transplant MAP of 106.56 mmHg (SD=16.51), with a mean reduction by 1.27 mmHg after transplantation (*P* = 0.758, paired two-tailed student “t” test) [Figure 4]. The average nadir creatinine reached in these patients was 1.16 mg% (SD = 0.24). The mean creatinine levels at the time of detection of TRAS was 1.61 mg% (SD = 1.84) and at 6 months follow up was 1.26 mg% (SD = 0.53), with a mean reduction by 0.35mg% (*P* = 0.291) [Figure 5]. One patient had reached a nadir creatinine of 1.6 mg% on the fifth post-operative day but remained asymptomatic. He was lost to follow-up and five and half years later, presented to us with high PSV and elevated serum creatinine. In this group of 18 patients, 3 had symptoms suggestive of TRAS. Of the 3, two had hypertension requiring more than 2 drugs. They underwent angiography and were only followed up, as they had insignificant stenosis.

**Figure 6:**
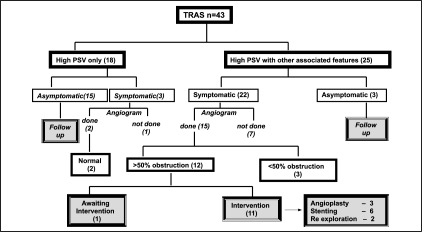
Flow chart showing the outcome of 43 patients with TRAS

The remaining 25 patients had other associated features on DUS apart from high PSV levels. Of these, 14 had a low resistive index, 6 had parvus et tardus pattern of flow and 5 had an intra-segmental dampening of flow recorded. Three patients had more than one associated feature observed in Doppler. Of these 25 patients, 3 were asymptomatic and did not require any further evaluation. Of the remaining 22, 15 required angiographic evaluation. The remaining 7 patients had hypertension well under control with two drugs and were followed up. Of the 15 who underwent angiographic evaluation, 3 had < 50% luminal narrowing, requiring only a close follow up. Of the remaining 12 patients, 11 were subjected to intervention in the form of angioplasty in 3, stenting in 6, and re-exploration in 2 patients. The remaining one patient is awaiting intervention. Of the two patients who were re-explored, one patient had extravasation of contrast at the site of anastomosis during angiogram and the other had a sharp angulation at the site of anastomosis, which required a revision of anastomosis. The flow chart in [Figure 6] represents the details of the 43 patients in our study.

**Figure 7:**
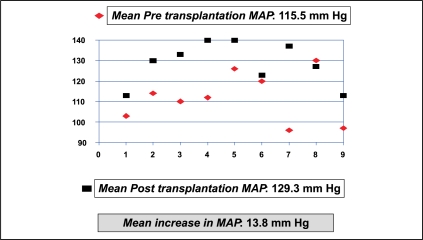
Mean arterial pressure before and after transplantation (n=10)

**Figure 8:**
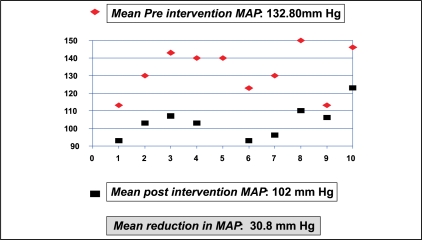
Mean arterial pressure before and after intervention (n=10)

**Figure 9:**
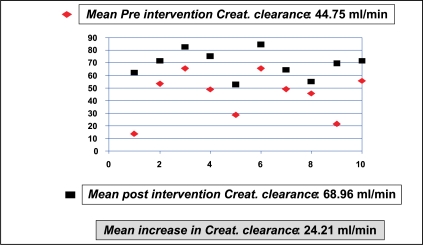
Graft function before and after intervention (n=10)

**Figure 10:**
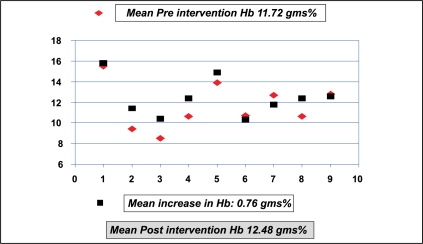
Hb status before and after intervention (n=10)

Of the 11 patients who had intervention, one expired in the immediate postoperative period after re-exploration, of urosepsis and disseminated intravascular coagulation. The data from the remaining 10 patients were analyzed. The MAP before and after renal transplantation was 115.50 mmHg and 129.30 mmHg with a difference of 13.8 mmHg (*P*=0.006) [Figure 7]. The MAP before and one week after intervention were 132.8 mmHg (SD = 13.22) and 102 mmHg (SD = 10.55), respectively, with a reduction in MAP by 30.8 mmHg and a SD of 13.181 (*P*=0.000) [Figure 8]. The mean creatinine clearance (Cockroft-Gault method) before and after intervention was 44.75 ml/min (SD=17.85) and 68.96 ml/min (SD = 10.56), respectively, with a mean increase by 24.21 ml/min (*P*=0.000) [Figure 9]. The mean pre- and post-intervention haemoglobin (Hb) levels were 11.72 gm% (SD = 2.14) and 12.48 gm% (SD = 1.76), with an average improvement in Hb status by 0.76 gm% (*P*=0.05) [Figure 10].

## DISCUSSION

Ischemia to the transplant kidney can occur due to various medical factors (both immune and drug related) and surgical causes including TRAS. It is important for transplant surgeons and the treating physicians to address this contentious issue. The host's immune response has been a major cause of premature allograft loss for many years. However, with the introduction of powerful immunosuppression in the last two decades, there has been a considerable reduction in the allograft loss due to rejection episodes.

Renal artery stenosis is one of the major vascular complications of renal transplantation, which usually presents with uncontrolled hypertension or unexplained renal dysfunction. Hypertension following renal transplantation can be multifactorial including chronic rejection, cyclosporine toxicity, use of corticosteroids, recurrent glomerulonephritis, native kidney disease or TRAS.[5]

With a high index of clinical suspicion and aided by DUS, TRAS can be diagnosed early and treated appropriately, with a reversal of post transplant hypertension and improvement in GFR. Wong *et al*. had reported a prevalence of 2.4% before and 12.4% after the introduction of routine DUS.[6] Investigators at University of Minnesota reviewed 2013 cases of adult kidney transplants performed between 1984 and 1998 and reported a prevalence of TRAS of 2.3%.[7] Mammen *et al*. from our institution had earlier reported an incidence of TRAS of 1.7%, when routine postoperative Doppler evaluation was not in vogue.[8] After the introduction of routine postoperative Doppler evaluation since 2002, we now report an incidence of 7.9% of TRAS, which is similar to that reported in other studies. In our present series, there has been a sudden increase in the number of TRAS patients detected in the last 3 years. The reasons could be multifactorial. A high index of clinical suspicion, the aggressiveness with which the patients are being evaluated for TRAS and different levels of surgical expertise might all play a role.

Renal artery stenosis is more common following transplants from deceased donors as compared to live donor transplants in most of the studies, suggesting the possibility of immunological factors and prolonged cold ischemia, playing a role.[9] However, our center mainly performs living related transplants and we had only 2 of the 34 deceased kidney recipients developing TRAS.

DUS is a well-accepted screening tool for assessment of renal vasculature. Even though DUS is observer dependent, it is easy to use, non-invasive, inexpensive and a highly reliable tool.[10] Moreover, it does not require radioactive tracers and is highly sensitive and specific.[11] The 18 patients (42%) in our study, who had only a high PSV documented in DUS, had a stable serum creatinine and blood pressure at the time of detection and during follow up even though the number of antihypertensive drug requirement increased postoperatively. Documentation of high PSV alone in DUS can only indicate that the patient requires a closer follow up. None of the 18 patients (42%) in our study with isolated finding of high PSV in DUS required any form of further intervention, further reinforcing the fact that it is mainly the clinical features which decide the need for further intervention.

The decision to do angiographic evaluation was largely based on clinical symptomatology and not merely based on the Doppler findings. The Doppler evaluation was only used as a guide to warn us of those patients requiring a closer monitoring or further evaluation including angiogram.

Many criteria are used to detect TRAS using DUS. PSV, intra-renal dampening of flow and resistive index are the important diagnostic parameters. Baxter *et al*. suggested a PSV of more than 250 cm/s to make a diagnosis of TRAS.[12] Patel *et al*. observed that for screening, a PSV of 250 cm/s had a poor specificity.[13] Mahesh Goel *et al*.[10] reported that of the 25 patients belonging to the high probability group with a PSV of more than 200 cm/s, 20 patients required additional investigations including angiogram, of whom, 13 required further interventions. In our series, of the 43 with a PSV of more than 200 cm/s, 25 had other associated anomalies detected in Doppler, and 17 underwent angiogram.

Sankari *et al*. from Cleveland clinic, in their study on long term outcomes of different treatment methods on 23 patients with TRAS observed that 16 out of 23 patients (75%) required angioplasty and 5 required surgical revascularization.[14] About 75% of them showed improvement with respect to hypertension and 69% had an overall improvement in allograft function. In our series, 10 of the 11 recipients who required intervention showed an overall improvement in blood pressure and renal function.

Morais *et al*. evaluated the role of Duplex Doppler sonography in diagnosing TRAS. In their study on 21 patients with suspected TRAS, they reported that a PSV of more than 200 cm/s, acceleration time of 0.1 s or higher in the renal or intra renal arteries and ratio of PSVs in the renal and iliac arteries of greater than 1.8 were accurate parameters to diagnose TRAS.[15] The role of magnetic resonance imaging in detection of TRAS is limited, with a high false positive rate. Luk *et al*. assessed the value of gadolinium enhanced subtraction magnetic resonance angiography (GD MRA) in seven patients with suspected TRAS. They concluded that GD MRA correlated with the gold standard digital subtraction angiography (DSA) and is a promising and non-invasive technique in detection of TRAS, particularly in patients with abnormal renal function.[16] However, Loubevre *et al*. in their study on 12 patients with clinical suspicion of TRAS compared DUS with MRA and DSA and concluded that MRA is of limited diagnostic value for making a diagnosis of TRAS because of a 75% incidence of false positive report with MRA.[17] The reason attributed for this very high false positive rate is a major intravoxel phase dispersion, which may occur due to either tortuosity of the vessel or because of a sharp angulation between the renal artery and the parent vessel. To conclude, Duplex Doppler sonography is an excellent method for screening patients suspected to have TRAS and can help in selecting those who should undergo arteriography.

## CONCLUSIONS

This article mainly highlights the need for increasing the awareness of this potentially curable problem. It reinforces the need for a high index of clinical suspicion and liberal use of non-invasive screening modalities, which may enable us to detect all haemodynamically significant stenosis. Moreover, an isolate finding of high PSV in DUS recommends a close follow-up, but does not necessarily warrant further intervention unless the other associated features are also present. Early detection and an appropriate treatment of TRAS could improve the overall allograft function.
